# The triad of physiological challenges: investigating the intersection of sarcopenia, malnutrition, and malnutrition-sarcopenia syndrome in patients with COPD - a cross-sectional study

**DOI:** 10.1186/s12890-024-02884-3

**Published:** 2024-02-05

**Authors:** M. Yogesh, Jenish Patel, Naresh Makwana, Mansi Mody

**Affiliations:** 1 Department of Community Medicine, Shri M P Shah Government Medical College, Jamnagar, Gujarat India; 2 Shri M P Shah Government Medical College, Jamnagar, Gujarat 361008 India

**Keywords:** Chronic obstructive pulmonary disease, Sarcopenia, Malnutrition-sarcopenia syndrome, Malnutrition

## Abstract

**Background:**

One of the most prevalent respiratory disorders in modern society is chronic obstructive pulmonary disease (COPD). Frequent comorbidities in patients with COPD are abnormal nutritional status and body composition variations. Malnutrition-sarcopenia syndrome, which occurs when the 2 conditions – malnutrition and sarcopenia – coexist, raises the risk of death more than either condition alone. The current study sought to determine the prevalence of malnutrition, sarcopenia, and malnutrition-sarcopenia syndrome in patients with COPD as well as the association between these diseases and the severity of COPD.

**Methods:**

The study was an analytical cross-sectional study conducted on hospitalized patients with COPD. The sample size of the study was calculated to be 160. A self-structured questionnaire was used to collect the data, containing sociodemographic characteristics, clinical profiles, anthropometric assessment, and bioimpedance indices. Sarcopenia was diagnosed with low muscle strength and muscle mass by the EWGSOP2 recommendations. Muscle mass is measured by BIA and muscle strength (Handgrip) was measured by a Hand Dynamometer. Assessment of the risk of malnutrition was performed using the Mini Nutritional Assessment-Short Form questionnaire and was confirmed by GLIM criteria. The COPD assessment test (CAT) tool determined the severity of the condition. For the data analysis, comparisons were made using Student’s t test and Mann–Whitney test in bivariate analysis. Multivariate logistic regression analyses were performed considering the outcomes of patients with COPD by CAT scores, prolonged length of stay, and hospital readmission 6 months after discharge.

**Results:**

The mean age of the participants was 48 ± 5 years. Approximately 61.9% were found to be sarcopenic. Approximately 45.6% of participants had malnutrition. Malnutrition sarcopenia syndrome was diagnosed in 32.5% of patients. The study analysis revealed that patients with COPD with malnutrition-sarcopenia syndrome had more than twice the odds of prolonged hospital stay, re-admission within 6 months, and higher CAT scores.

**Conclusion:**

The study revealed a high prevalence of sarcopenia, malnutrition, and malnutrition sarcopenia syndrome in patients with COPD. These conditions were found to be statistically significant with prolonged length of stay, re-admission within 6 months, and CAT scores. The findings highlight the importance of addressing these conditions as part of the management of the patients.

## Background

One of the most prevalent respiratory disorders in modern society is chronic obstructive pulmonary disease (COPD). It is currently the fourth most common cause of mortality worldwide and is expected to move up to the third spot soon [1]. The global prevalence of COPD is estimated to be 12%, with studies showing a higher prevalence among older adults compared to younger populations under age 50 (21.38% vs 5.28%) [1].

An inflammatory disorder, COPD [2], in addition to obstructing airflow, is accompanied by numerous systemic symptoms, which have a significant impact on prognosis and treatment costs. The most frequent comorbidities in patients with COPD are abnormal nutritional status and changes in body composition, which have a significant detrimental effect on prognosis (increased risk of COPD exacerbations, depression, or mortality) [3–6]. According to data from several studies, 30–60% of patients with COPD are undernourished [7–9], 20–40% have reduced muscle mass [3, 4], and 15–21.6% have sarcopenia [10, 11]. Malnutrition and sarcopenia both have a detrimental effect on how COPD develops: they reduce exercise tolerance, raise the chance of hospitalization, and lower quality of life [4, 10, 12].

Malnutrition-sarcopenia syndrome (MSS), which occurs when the 2 conditions coexist, raises the risk of death more than either condition alone [13]. Older adults are especially vulnerable to these issues because of changes brought on by aging [14].

A healthy adult burns between 36 and 72 cal per day just to breathe. Severe blockage in the respiratory tract and COPD may require almost 5 times as much energy for this function. The rehabilitation process in undernourished older people with COPD may be positively influenced by a dietary intervention (i.e., higher protein delivery) targeted at increasing muscle mass and enhancing respiratory force, physical capacity, general health condition, and quality of life [6, 15, 16].

Malnutrition refers to deficient intake or uptake of calories, proteins, or other nutrients due to an underlying health condition or inadequate diet. It leads to altered body composition and diminished physical and mental function and Sarcopenia is the degenerative age-associated loss of skeletal muscle mass and strength. It results in reduced mobility, increased fatigue, and a higher risk of adverse events [17, 18].

Physical frailty is characterized by progressive diminishing of strength, endurance, and reduced physiological function. It shares overlapping features with sarcopenia including decreased muscle mass and weakness [19]. Frailty often coincides with the presentation of sarcopenia in older adults and further elevates the risk of adverse health outcomes and mortality [20]. Assessing frailty indicators in parallel with sarcopenia can aid in gauging severity and prognosis. While malnutrition and sarcopenia may co-occur, particularly in older patients, they have distinct physiological mechanisms and etiologies. Reduced food intake, impaired digestion and disease-related hypermetabolism can precipitate malnutrition resulting in muscle wasting from nutritive deprivation [21].

Recognizing malnutrition and sarcopenia as distinct conditions with divergent pathogenesis is pivotal, as it consequently impacts prognosis and treatment approach. While nutritional intervention forms the cornerstone of malnutrition management, dietary measures like protein supplementation have demonstrated limited effectiveness in reversing sarcopenia progression [22]. A multimodal strategy including exercise, testosterone and other pharmacological options tailored to counteracting muscle deterioration offers greater promise for combating sarcopenia [23]. Identifying coinciding malnutrition and sarcopenia and delineating key drivers allows optimized, directed treatment planning rather than a one-size-fits-all approach. This underscores the relevance of our study in determining the prevalence of both these conditions among patients with COPD using recent standardized definitions.

While numerous studies have examined the prevalence of sarcopenia or malnutrition in people with COPD, there is limited literature available that used recent diagnostic standards (GLIM and EWGSOP2) [24–28]. Nevertheless, none of those studies addressed both illnesses. The current study sought to determine the prevalence of malnutrition, sarcopenia, and malnutrition-sarcopenia syndrome in patients with COPD as well as the association between these diseases and the severity of COPD.

## Methodology

The study was a hospital-based analytical cross-sectional study conducted in a tertiary care hospital over hospitalized patients during the period of January 2023–March 2023. Adult hospitalized patients who had been diagnosed with COPD had an absence of acute exacerbation of COPD and gave informed, written consent to participate in the study were included. Participants who had active malignancy or contraindications for body composition analysis with a bioimpedance method (like those with metal implants, implanted cardiac devices, or oedemas), were critically ill or could not respond, and those who did not consent to participation were excluded from the study [29].

### Sample size

Utilizing the prevalence estimate (10%) for malnutrition sarcopenia syndrome [29], the sample size was computed to be 138 at a 4% precision and 97% confidence level using the following sample size formula: ZP(1-P)/d^2,^ where Z is the Z statistic for a level of confidence (3.84), P is the expected prevalence and d is the precision level. This was projected as a conservative estimate, as the prevalence was anticipated to be higher in the presence of COPD. Based on this, we analysed a sample size of 160, which was slightly more than the calculated value, i.e., 138. All the participants were given instructions about the study before the start of the study. Good clinical care were performed. All the participants were given instructions about the study before the start of the study. Written informed consent was obtained in their Vernacular Language. Ethical approval from the institute (Shri M P Shah Government Medical College, Jamnagar, Gujarat, India) was obtained before the start of the study. (REF No:37/01/23).

### Data collection tool

Electronic medical records were used to obtain sociodemographic data such as age, sex, socioeconomic status, and occupation. Next, skeletal muscle % was measured using a Bio-Electrical Impedance analysis machine (Omron Body composition monitor, Model HBF-702 T). Moreover, a hand dynamometer was used to measure hand grip strength.

#### Assessment of risk of malnutrition

Assessment of the risk of malnutrition was performed using the Mini Nutritional Assessment-Short Form questionnaire (MNA-SF) and GLIM criteria. If at least 1 phenotypic and at least 1 etiologic criterion out of the following were fulfilled, the diagnosis of malnutrition was confirmed as recommended by the GLIM experts [17].

Phenotypic criteria: (1) Unintentional body weight loss: the loss of > 5% habitual body mass within the past 6 months or the loss of > 10% in more than 6 months. (2) Low body mass index (BMI): < 20 kg/m^2^ in subjects below 70 years and < 22 kg/m^2^ in individuals 70 years or older. (3). Muscle mass was assessed based on the measurement of appendicular lean mass (ALM) and calculation of the ALM index, which represents the ratio of ALM (kg) to the square of height (m^2^). An ALM index below the cut-off points for the Polish population (5.6 kg/m^2^ in women and 7.4 kg/m^2^ in men) was indicative of low muscle mass (LMM). The appendicular lean mass was measured with the electrical bioimpedance method (BIA) (Omron Karada scan, 702 T).

Etiologic criteria: (1) Reduced food intake or assimilation was recognized in subjects declaring any reduction in food intake within the past 3 months in the MNA-SF questionnaire. (2) A significant disease burden/inflammatory condition was recognized in all participants with a COPD diagnosis.

#### Assessment of sarcopenia

To diagnose sarcopenia**,** the following measurements were taken:

- Muscle mass was measured using a bioelectrical impedance analysis (BIA) machine, and a skeletal muscle mass index (SMMI) of less than 7.0 kg/m^2^ for men and less than 5.5 kg/m^2^ for women can be used to diagnose sarcopenia using BIA. The cut-off points for low muscle strength and muscle mass followed the EWGSOP2 recommendations [18].
- Muscle strength was measured using handgrip strength: To perform the test, the person being tested holds a handgrip dynamometer and squeezes it with maximum effort for a few seconds. The test was repeated three 3 times on each hand, and the highest value was used for analysis. The cut-off value for diagnosing sarcopenia using handgrip strength varies depending on factors such as age, sex, and population studied. However, a handgrip strength of less than 27 kg for men and less than 20 kg for women is commonly used as a cut-off value for diagnosing sarcopenia [18].

#### Assessment of severity of Copd

The COPD assessment test (CAT) is a tool that can help in communicating the severity of the condition [30]. The CAT asks questions about 8 areas, prompting the patient to assign a score ranging from 0 to 5 for each area. A score of 0 meant there was no impairment in that area. A score of 5 indicated severe impairment. The overall score ranged from 0 to 40. Higher scores indicate that COPD has a greater impact on overall health and well-being. Generally, the Global Initiative for Obstructive Lung Disease (GOLD) guidelines suggest using a CAT score of 10 or above to indicate symptomatic COPD [30].

The MNA-SF and CAT were interviewer-administered with extensive support (medical students and resident doctors) from our multilingual study team to promote patient understanding and reliable data collection.

### Statistical analysis

All data were transferred and sorted in MS 2006. Descriptive statistics were also calculated. The mean and standard deviation for parametric quantitative variables, the median and interquartile range for nonparametric variables, and absolute and relative frequency for categorical variables were incorporated. The Kolmogorov–Smirnov method was used to test the quantitative variable normality. For the data analysis, patients with probable sarcopenia and those with sarcopenia were grouped and compared to nonsarcopenic patients by Student’s t test and Mann–Whitney test in bivariate analysis. Multivariate logistic regression analyses were performed considering the outcomes of patients with COPD by CAT scores, prolonged length of stay (LOS) (categorized by a median of 10 days considering its data distribution in our sample), and hospital readmission 6 months after discharge. For multivariate regression analysis, CAT score was dichotomized into a binary categorical variable with a cut-off of 10, as this threshold indicates symptomatic COPD (CAT < 10 versus CAT ≥10).” based on prior literature [31, 32]. In the multivariate logistic regression models, we constructed three independent models, considering sarcopenia, malnutrition, and MSS, as independent predictors of the outcome. The dependent variables were: prolonged length of stay (> 10 days), readmission within 6 months, and COPD assessment test (CAT) scores. The independent variable in each model was one of the three predictors: malnutrition, sarcopenia or MSS. Potential confounders like age, sex, BMI were adjusted for in the models based on their significance (*p* <  0.2) in bivariate analyses comparing patients with and without the conditions. BMI was ultimately excluded due to its co-linearity with malnutrition. The purpose was to determine the association of malnutrition, sarcopenia and MSS individually with the outcomes while controlling for potential confounding factors. The entire analysis was performed in SPSS 26.0 software, and *p* values < 0.05 were considered statistically significant. *p* <  0.001 was considered highly statistically significant.

## Results

**Fig. 1:**
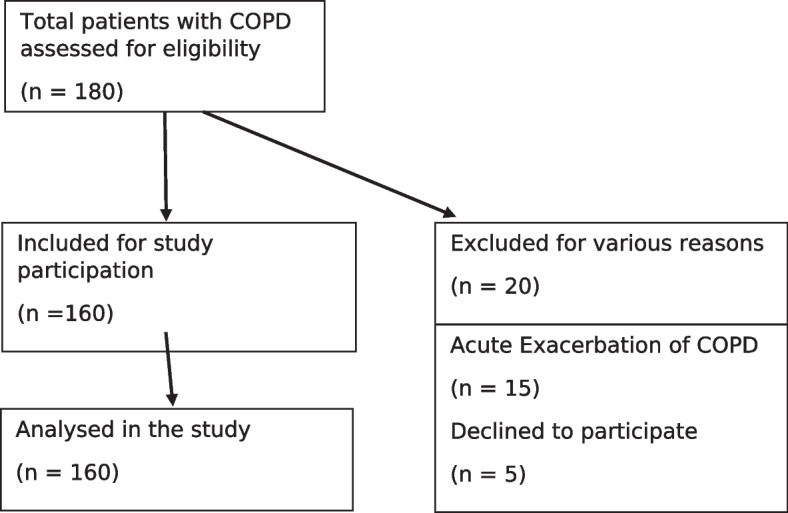
Participants' enrollment in the study

Of 180 eligible patients, 160 met the inclusion criteria. Of the remaining 20 patients, 5 declined to participate due to reasons such as being in a hurry for discharge (*n* = 2), hesitation about the bioimpedance test (*n* = 1), or general disinterest (*n* = 2). The other 15 patients were excluded due to acute exacerbation of illness (Fig. 1).

A total of 160 patients were included in the current analysis with a mean age of 48 ± 5 years. A total of 56.2% (*n* = 90) were males, and 43.8% (*n* = 70) were females.

The means of actual body weight and BMI were noted to be 50 ±  8 kg and 23 ± 5 ^kg/m2^, respectively. In the analysis involving a total of 160 patients, 73 (45.6%) were identified as having malnutrition (A), while 99 (61.9%) were found to be sarcopenic (B). The intersection of patients with both malnutrition and sarcopenia (A∩B) was determined to be 52 (32.5%) individuals, and 40 (25%) were patients with COPD without malnutrition or sarcopenia.

Utilizing the formula for overlapping sets, where A + B - (A∩B) equals the total number of patients (n), the calculation was resolved as 160 = 73 + 99–52. Further analysis revealed that 21 (13.1%) patients had only malnutrition (A - A∩B), and 47 (29.4%) patients exhibited only sarcopenia (B - A∩B). Therefore, the comprehensive breakdown is as follows: 52 (32.5%) patients with both malnutrition and sarcopenia, 21 (13.1%) patients with exclusive malnutrition, and 47 (29.4%) patients with exclusive sarcopenia, resulting in a total of 120 (75%) individuals across these categories.

Table 1 shows the comparison of anthropometric variables and bioimpedance characteristics of subgroups of sarcopenic and nonsarcopenic patients. Patients with sarcopenia had statistically and clinically significant lower body weight and BMI than nonsarcopenic patients (*p* <  0.05). Their body composition also significantly varied since the participants with sarcopenia had relatively lower fat-free mass (FFM) and SMMI than participants without sarcopenia. However, they had comparatively higher body fat % than nonsarcopenia patients. Nevertheless, patients with sarcopenia had remarkably lower hand grip strength and MNA-SF scores than patients without sarcopenia.

**Table 1:** Comparison of anthropometric and bioimpedance indices of patients grouped according to the diagnosis of sarcopenia

Variables	Non Sarcopenic Patients (*n* = 61)	Sarcopenic Patients (*n* = 99)	*p* Value
Age (years)	47 ±14	47.85 ±15	0.956
Body Weight (kg)	59.41 ± 10.5	48.64 ± 11	< 0.001
Height (cm)	160 ± 9.62	157 ±10.18	0.109
BMI (kg/m^2^)	23.37 ± 4.7	18.86 ± 5.11	< 0.001
Body Fat %	21.79 ± 7.2	24.32 ± 7.5	0.036
**Fat Free Mass (FFM)(kg)**	23.5 ±4.6	18.2 ±4.5	< 0.05
**MNA-SF**	11 ± 2.5	8 ± 2.4	< 0.05
**SMMI (kg/m**^**2**^**)**	19.98 ± 4.19	5 ± 0.8	< 0.001
**Hand Grip (kg)**	13.13 ±13.07	7.67 ± 6.7	< 0.001

*p* value < 0.05- statistically significant. *p* value < 0.001 - highly statistically significant

Table 2 compares the anthropometric variables and bioimpedance characteristics between patients with and without malnutrition. Patients suffering from malnutrition were significantly taller, older, and had statistically and clinically significantly lower body weight and BMI than patients with no malnutrition. Their body composition also significantly varied since the participants with malnutrition had relatively lower fat-free mass (FFM) and SMMI than participants without malnutrition. Additionally, participants with malnutrition had remarkably lower hand grip strength and MNA-SF scores than the other subgroups.

**Table 2:** Comparison of anthropometric and bioimpedance indices of patients, classified on the basis of the presence and absence of malnutrition

Variables	Patients without Malnutrition (*n* = 87)	Patients with Malnutrition (*n* = 73)	*P* Value
Age	44 ± 14.83	50 ± 15.28	0.025
Body Weight	61 .02 ± 10.40	43 .28 ± 5.87	< 0.001
Height	156 ± 10	162 ± 8	< 0.001
BMI (kg/m^2^)	24 ± 4.32	16.5 ± 2.38	< 0.001
Body Fat %	24 ± 8.41	22 ±6	0.063
FFM (kg)	22.9 ±5.2	15.5 ±4.7	< 0.05
MNA-SF	12.5 ±2.5	9.2 ±3.2	< 0.05
SMMI (kg/m^2^)	14.62 ±8.34	6.79 ± 4.35	< 0.001
Hand Grip (kg)	11.95 ± 11.08	7.12 ± 7.9	0.008

*p* value < 0.05- statistically significant. *p* value < 0.001 - highly statistically significant

Table 3 shows the comparison of anthropometric variables and bioimpedance characteristics between patients with COPD with MSS and patients with COPD without MSS. Patients with MSS had statistically and clinically significant lower body weight and BMI when compared to non-MSS patients, simultaneously having taller heights. Their body composition also significantly varied since the participants with MSS had relatively lower fat-free mass (FFM), MNA-SF score and SMMI than participants without MSS. Moreover, patients with MSS had remarkably lower hand grip strength than patients without MSS.

**Table 3:** Comparison of anthropometric and bioimpedance indices of study participants grouped on the basis of the presence and absence of MSS

Variables	Non-MSS Patients (*n* = 108)	MSS Patients (*n* = 52)	*P*- Value
Age (years)	49.47 ±15	44.97 ± 16	0.108
Body Weight (kg)	59.51 ± 10.75	42 .23± 5.29	< 0.001
Height (cm)	157.67 ± 10.3	162.1 ± 8.5	< 0.003
BMI (kg/m^2^)	24.07 ± 4.61	16 .15± 2.361	< 0.001
Body Fat %	23.78 ± 8.26	22.71 ± 6	0.386
FFM (kg)	22 ±3.9	17 ±4.2	< 0.05
MNA-SF	12.25 ±3.2	9 ±2.4	< 0.05
SMMI (kg/m^2^)	14.79 ± 7.89	4.98 ± 0.919	< 0.001
Hand Grip (kg)	11.96 ± 12.14	6.16 ± 5.34	< 0.001

*p* value < 0.05- statistically significant. *p* value < 0.001 - highly statistically significant

Table 4 shows the association between malnutrition, sarcopenia, and MSS with prolonged hospital stay, readmission within 6 months, and CAT scores of COPD, which were statistically significant. The present study found that malnutrition had odds ratios of 2.35 (*p* = 0.024) and 2.45 (*p* = 0.009) for prolonged length of stay and readmission within 6 months, respectively. This implied that patients with malnutrition were 2.35 times more likely to have a prolonged length of stay and 2.45 times more likely to be readmitted within 6 months than patients without malnutrition. An odds ratio of 2.17 (*p* = 0.021) for higher CAT scores in COPD was also found in participants with malnutrition, which meant that patients with malnutrition were 2.17 times more likely to have a higher CAT score than those who did not suffer from malnutrition. Similar results were obtained when the association of sarcopenia and MSS was found with a prolonged LOS, readmission within 6 months, and higher CAT scores. Sarcopenic patients with COPD had 3.68 higher odds (OR = 3.68, *p* = 0.004) of prolonged LOS, 3.92 higher odds (OR = 3.92, *p* = 0.001) of being readmitted within 6 months, 2.80 higher odds (OR = 2.8, *p* = 0.05) of having higher CAT scores than non-sarcopenic patients. Compared to patients with COPD without MSS, those with MSS had 2.7 times the odds (OR = 2.7, p = 0.004) of prolonged hospital stay, and 2 times the odds (OR = 2, *p* = 0.04) of readmission within 6 months. Patients with COPD with MSS had 2.1 higher odds (OR = 2.1, *p* = 0.029) of having elevated CAT scores compared to those without MSS.

**Table 4:** Association of sarcopenia, MSS, and malnutrition with clinical outcomes of patients with COPD

Predictors	Malnutrition		Sarcopenia		MSS	
	OR	*p* Value	OR	*p* Value	OR	*p* Value
**Prolonged LOS (> 10 Days)**	2.35	0.024	3.684	0.004	2.721	0.004
**Re-Admission within 6 months**	2.453	0.009	3.916	0.001	1.98	0.04
**CAT score ≥ 10**	2.162	0.021	2.798	0.005	2.08	0.029

*p* value < 0.05- statistically significant. *p* value < 0.001 - highly statistically significant

## Discussion

The key finding of this study was the high prevalence of sarcopenia (61.9%), malnutrition (45.6%) and malnutrition-sarcopenia syndrome (32.5%) among hospitalized patients with COPD.

- Comparing the study findings with previous research, Smith et al. [33] reported that malnutrition affects up to 35% of individuals with COPD, which is lower than the prevalence found in the current study. Patel et al. [34] found a lower prevalence of malnutrition in patients with COPD at 17%, which is significantly lower than the prevalence found in the current study. However, it is important to note that the sample size of the study in the discussion was smaller than that of the current study, which might be an explanation for the discrepancy. Differing inclusion/exclusion criteria and patient populations, use of different diagnostic criteria and screening tools, underlying nuances in study design, sampling methods may also contribute to the difference.

Limpawattana et al. [35] reported that the prevalence of sarcopenia in COPD men older than 40 years of age varies from 20 to 40% depending on the age of the studied population, gender, and other factors. This is lower than the prevalence found in the current study. Collins et al. [6] reported that malnutrition, sarcopenia, and frailty are common in patients with COPD and are often associated with periods of elevated systemic inflammation and the presence of pulmonary cachexia.

The study analysis revealed that patients with COPD with malnutrition had more than twice the odds of prolonged hospital stay, readmission within 6 months, and higher CAT scores. Comparing these findings with previous studies, a study conducted on adult COVID-19 patients found that patients with malnutrition had a 76% increased risk of mortality and 105% higher chances of longer hospital length of stay [36]. Riccardo et al. found that the risk of nutritional deficiency at admission was strongly associated with a prolonged hospital stay among ambulatory adult patients [37]. Furthermore, Yogesh et al. reported that malnutrition was associated with a significantly higher risk of the combined endpoint of readmissions or death within 7 days [38].

Hence, the findings of the present study are consistent with previous studies that have shown that malnutrition is associated with adverse outcomes in hospitalized patients. The present study adds to the literature by showing that malnutrition is also associated with higher CAT scores in patients with COPD. The results of this study suggest that nutritional interventions may be beneficial in reducing the risk of adverse outcomes in hospitalized patients with COPD.

The study analysis revealed that patients with COPD with malnutrition-sarcopenia syndrome had more than twice the odds of prolonged hospital stay, re-admission within 6 months, and higher CAT scores.

These results are in line with other research that found a connection between MSS and extended duration of stay and readmission. For instance, MSS was discovered in research by Sousa et al. [39] to be a predictor of worse outcomes in hospitalized patients. A deterioration in nutritional status during the first week of hospitalization was linked to a higher risk of an extended duration of stay and readmission, according to impactful research by Correia et al. [40]. In a similar vein, Cruz-Jentoft et al.’s study [18] also concluded the same, i.e., hospitalised older adults with sarcopenia and malnutrition had a greater risk of readmission and mortality. These findings indicate that MSS should be taken into account in clinical practice, as it may affect patient outcomes and healthcare expenses.

While malnutrition and MSS may exacerbate systemic inflammation in COPD, sarcopenia appears uniquely positioned to also directly impair respiratory muscle function. Multiple studies have found dyspnea and decreased lung capacity in patients with COPD correlate more strongly with reductions in respiratory muscle mass and strength than limb muscle wasting (Lewis et al. 2007; Nishimura et al. 2002) [41, 42] . This may be due to the heightened metabolic demands of breathing placing the respiratory muscles under consistent strain (Ottenheijm et al. 2005) [43].

Furthermore, research indicates cytokines like TNF-α and IL-6 spike earlier in the disease course in respiratory vs. limb muscles of patients with COPD (Fermoselle et al. 2012) [44]. We speculate this early, localized inflammatory response could be an initial trigger driving dysfunction and atrophy preferentially in the respiratory musculature.

Together, these factors may explain why sarcopenia associated more robustly than MSS or malnutrition alone with COPD severity indicators like CAT scores in our analysis. Screening for and mitigating sarcopenia progression through interventions could help maintain lung capacity and reduce COPD-related hospitalizations. Further mechanistic studies are warranted to fully illustrate sarcopenia’s direct contributions to COPD pathophysiology.

Limitations of the study include its cross-sectional design - the study design limits its ability to establish a cause-and-effect relationship between sarcopenia, malnutrition, and malnutrition sarcopenia syndrome in patients with COPD. Longitudinal studies are needed to determine the temporal relationship between these conditions and their impact on clinical outcomes. Moreover, the study was conducted at a single centre, which may limit the generalizability of the findings to other populations, ethnicities or healthcare settings. Further research involving multiple institutions and diverse patient populations is warranted to validate our results. The sample size of the study might not be sufficient to provide a comprehensive representation of the entire COPD population. Larger sample sizes could provide more robust and reliable results. Furthermore, the study relied on the participation and consent of patients, which may introduce selection bias. Patients who were unwilling or unable to participate might differ systematically from those who did, potentially affecting the prevalence estimates.

Nevertheless, the study suggests certain recommendations to be included in the therapeutic measures undertaken. They are enlisted as follows:

- Early Screening: Routine screening for sarcopenia, malnutrition, and malnutrition sarcopenia syndrome should be implemented in the management of patients with COPD. Early identification of these conditions can allow for timely interventions and potentially improve patient outcomes.
- Multidisciplinary Approach: A multidisciplinary approach involving healthcare professionals such as pulmonologists, dieticians, and physiotherapists should be adopted to address the complex interplay between COPD, sarcopenia, and malnutrition. Collaborative efforts can help develop tailored treatment plans and optimize patient care.
- Longitudinal Studies: Future research should focus on longitudinal studies to establish the temporal relationship between sarcopenia, malnutrition, malnutrition sarcopenia syndrome, and clinical outcomes in patients with COPD. This will provide a better understanding of the progression and impact of these conditions over time.
- Larger Sample Sizes: Conducting studies with larger sample sizes can enhance the generalizability and statistical power of the findings. Including diverse patient populations from multiple centres would contribute to a more comprehensive understanding of the prevalence and impact of sarcopenia, malnutrition, and malnutrition sarcopenia syndrome in COPD.
- Intervention Trials: Investigating the effectiveness of targeted interventions, such as exercise programs, nutritional support, and pharmacological therapies, in managing sarcopenia, malnutrition, and malnutrition sarcopenia syndrome in patients with COPD is warranted. These trials can guide evidence-based interventions and improve patient outcomes.

## Conclusion

Our study revealed a high prevalence of sarcopenia, malnutrition, and malnutrition sarcopenia syndrome in patients with COPD. These conditions were found to be statistically significant with prolonged length of stay, re-admission within 6 months, and CAT scores. The findings highlight the importance of addressing these conditions as part of the comprehensive management of patients with COPD.

## Data Availability

The datasets generated and/or analysed during the current study are not publicly available to protect the privacy of the study participants but are available from the corresponding author on reasonable request.

## Abbreviations

COPD: Chronic obstructive pulmonary disease
MSS: Malnutrition–Sarcopenia Syndrome
GLIM: Global Leadership Initiative on Malnutrition
EWGSOP2: European Working Group on Sarcopenia in Older People 2
MNA-SF: Mini Nutritional Assessment-Short Form
BMI: Body Mass Index
ALM: Appendicular Lean Mass
LMM: Low Muscle Mass
BIA: Bioimpedance Analysis
SMMI: Skeletal Muscle Mass Index
CAT: COPD Assessment Test
GOLD: Global initiative for Obstructive Lung Disease
LOS: Length of Stay
HGS: Hand-Grip strength
FFM: Free Fat Mass
